# In-Office Power Bleaching for the Aesthetic Management of Dental Fluorosis

**DOI:** 10.7759/cureus.59806

**Published:** 2024-05-07

**Authors:** Manoj Chandak, Paridhi Agrawal, Abhilasha Dass, Jay Bhopatkar

**Affiliations:** 1 Conservative Dentistry and Endodontics, Sharad Pawar Dental College and Hospital, Datta Meghe Institute of Higher Education and Research, Wardha, IND

**Keywords:** vital tooth bleaching, aesthetics in dentistry, dental bleaching, bleaching agents, fluorosis

## Abstract

Fluorosis is a widespread condition that is endemic and found in approximately 25 nations worldwide. It manifests as dental fluorosis, an inherited enamel imperfection resulting from excessive fluoride exposure during tooth development. This condition can lead to varying degrees of tooth discoloration, often requiring aesthetic correction. Bleaching represents one of the treatment approaches for such instances, with in-office power bleaching being a technique that comprises the clinical implementation and triggering of bleaching agents using light to expedite the procedure. This case report outlines the successful aesthetic revision of moderate dental fluorosis through power bleaching, obviating the demand for intrusive procedures. It underscores the efficacy and conservative nature of in-office power bleaching to address tooth discoloration associated with extensive fluorosis.

## Introduction

Fluorine plays an essential role in the mineralization of tooth and bone. Optimal levels of fluoride consumption produce an "anti-cariogenic effect" on teeth [1], with the World Health Organization (WHO) recommending a drinking water fluoride level of "1.5 ppm" [2]. However, excessive fluoride intake beyond optimal levels could result in systemic and dental fluorosis. Studies have revealed a favorable association between the risk of dental fluorosis and polymorphisms in genes such as the calcitonin receptor gene, estrogen receptor, and collagen type 1 alpha 2 [3], indicating that genetic factors may affect a person's susceptibility to the condition. An acquired flaw in dental enamel that arises from repeated exposure to high fluoride concentrations during tooth formation is known as dental fluorosis [4]. It happens when fluoride is present in close proximity to the developing tooth germ during the secretory and/or maturation phase of enamel production. Globally, fluorosis is widespread in approximately 25 countries [5].

Tooth discoloration, whether accompanied by malformation or not, can significantly impact an individual's appearance, social interactions, and overall quality of life [6]. Consequently, the management of stained teeth has become increasingly important in the field of cosmetic dentistry. Bleaching represents a non-invasive option for altering the shade of a tooth aesthetically. Vital teeth can be bleached using over-the-counter products, including in-office bleaching, dentist-supervised home bleaching, or self-bleaching [7]. In-office bleaching uses hydrogen peroxide (HP) at dosages ranging from 15% to 38%, which is administered right away to the exteriors of the tooth. To fasten the procedure, the primary chemical agent may be triggered via lasers, quartz halogen, ultraviolet units, plasma arc, or light-emitting diode, a technique usually referred to as "power bleaching" [8]. The application of activation methods through light results in reduced treatment times and mitigates adverse effects associated with bleaching materials [7]. The scarcity of reports on such treatment within our region underscores the need to emphasize the management of tooth staining caused by fluorosis through in-office bleaching.

## Case presentation

An 18-year-old female presented to the Department of Conservative Dentistry and Endodontics with a chief complaint of stained teeth in the upper front region of the jaw. The patient could not recall the exact duration since the discoloration began but noted that it had persisted for a long time and had progressively worsened. The patient did not report any pain or sensitivity related to the stained teeth. When questioned about the appearance of similar discoloration in her family or other residents in her native village, she mentioned that her younger brother and other residents also exhibited similar discoloration of their teeth.

**Figure 1:**
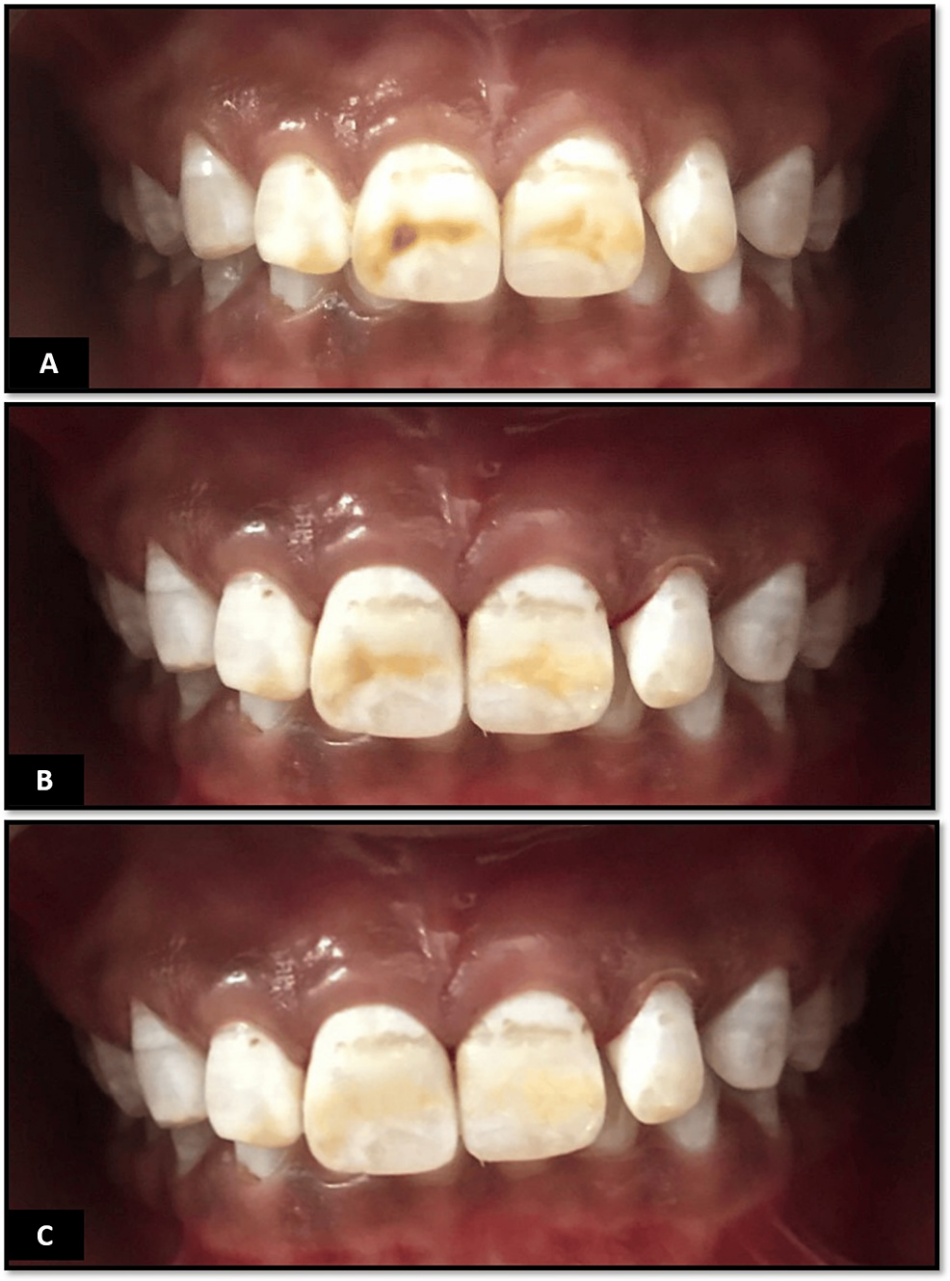
Clinical images (A) Pre-operative image. (B) Image after the first sitting of bleaching. (C) Image after the second sitting of bleaching

During intraoral examination, red to brown discoloration of the enamel was noted till the premolars of both arches. The discoloration displayed a bidirectional and proportional pattern, with few teeth exhibiting pitted surfaces. The severity of discoloration was pronounced on the upper arch juxtaposed with the lower arch (Figure 1A).

Nerve sensibility testing of the discolored teeth was performed using electric pulp testing and cold, revealing the usual pulp response in all teeth. The medical history given by the patient did not contribute to the diagnosis. After reviewing clinical observations and the patient's medical history, a diagnosis of moderate dental fluorosis was confirmed using Dean's fluorosis index [9].

The patient was presented with many treatment alternatives, including microabrasion, composite veneers, and in-office power bleaching. The patient elected to undergo "in-office power bleaching" after obtaining informed permission. The initial step involved assessing the tooth color using the Vita shade guide and capturing pre-treatment clinical photographs. Subsequently, all the anteriors of both arches were polished using a pumice and a rubber cup. To protect surrounding teeth and delicate tissues, a rubber dam along with a gingival barrier was applied. The patient wore protective eyewear throughout the procedure.

A 35% HP gel (Pola Office, SDI Ltd, Australia) was then employed to the anterior of the upper arch. The chemical agent was triggered using a power unit (Bluedent 12 BL bleaching unit) for 15 minutes. After each activation cycle, the gel was wiped out with gauze, and the same approach was redone two times more. Upon completion of the protocol, all the employed chemical was suctioned out, and the teeth were thoroughly cleaned using water. The patient returned for a second visit after one week, during which the same approach was resumed again. At the end of the second visit, a total of six cycles of bleaching including the first visit were completed. The second visit concluded with satisfactory expression from the patient, with a lightening of the color of the tooth. Post-treatment shade was recorded, and clinical pictures were obtained at every follow-up (Figure 1B, 1C).

Following the end of the procedure, the possibility of further aesthetic enhancement through composite veneers was discussed with the patient. However, she expressed satisfaction with the look of her teeth and declined the veneers. Considering potential post-bleaching sensitivity, she was prescribed a desensitizing mouthwash. Subsequent follow-up appointments were scheduled at one, three, and six months. During these appointments, the patient reported no rebound effect, pain, or sensitivity linked with the bleached teeth.

## Discussion

Dental fluorosis often engenders an unsatisfactory self-image and a sense of discontent in affected individuals, with the degree of dissatisfaction correlating with the severity of fluorosis [10,11]. Tooth discoloration commonly prompts patients to seek treatment, and various aesthetic correction modalities are employed, including resin infiltration, bleaching, microabrasion, full crowns, and veneers. The conservative treatment options such as resin infiltration, bleaching, and microabrasion cause no significant impact on the physical properties of teeth when compared to the substantial tooth structure loss that occurs following macroabrasion, megabrasion, veneers, or crown preparation [10]. The degree of fluorosis determined according to various indices, for example, using Dean's fluorosis index, determines which treatment is best [12].

Power bleaching is one non-invasive method that can significantly improve the appearance of stained teeth and may not require additional treatment. If bleaching is ineffective, direct or indirect restorative methods like resin infiltration or composite veneers can be used. Still, they come with a price: they are costly, time-consuming, invasive, and damaging to the tooth's natural tissue [13]. Therefore, power bleaching might provide an advantageous outcome involving minimizing the extent of enamel and dentin removal required for composite veneers [14]. Research by Jarad et al. has demonstrated that bleaching prior to the placement of direct composite veneers significantly influences the concluding tone of the veneer. This factor also depends on the restoration's thickness or shade [15].

Direct composite veneers, direct composite laminate veneers, indirect composite veneers, or ceramic veneers could have been considered for the upper anterior following power bleaching in the presented case. However, the patient opted against this option, expressing satisfaction with the outcome of the bleaching treatment. Resin infiltration has emerged as a recent and effective conservative technique in managing dental fluorosis. This technique involves conditioning hydrochloric acid-conditioned fluorotic opacities before adding low-viscosity resin. Research has shown that the masking effect of resin infiltration by prior in-office power bleaching was increased on fluorotic opacities (Schoppmeier et al., 2016). In order to enhance the aesthetic results of resin infiltration, power bleaching may be a useful pre-treatment program [16].

Concerns have been expressed about the increase in surface and intra-pulpal temperatures associated with light-activated vital bleaching methods used in offices. Sulieman et al.'s research found that, with the exception of laser-based lamps, the increase in intra-pulpal temperature brought on by different lights (such as plasma arc lamps, xenon-halogen lamps, and conventional halogen lamps) stayed below the crucial threshold of 5.5°C [17]. One common adverse effect of bleaching methods is post-bleaching sensitivity, which is mostly caused by peroxide percolating through the dentin to the pulp. According to Moghadam et al., 42.9% of patients had sensitivity following at-home bleaching, whereas 57.1% had sensitivity after power bleaching [18]. Furthermore, Kabil et al. examined how a "descending light intensity protocol" affected post-bleaching sensitivity, finding that it did so at a lower level than the traditional "high light intensity protocol" [19]. To lessen post-bleaching sensitivity, desensitizing chemicals could be used [20].

## Conclusions

In-office bleaching emerges as a non-invasive and highly effective method for enhancing the aesthetics of teeth afflicted by severe fluorosis and discoloration. This treatment option offers a straightforward solution and serves as a valuable preliminary step for subsequent cosmetic procedures, whether direct or indirect restorations, and facilitates optimal resin penetration. Thus, in comparable scenarios, in-office bleaching presents itself as a versatile and advantageous approach to address both immediate cosmetic concerns and pave the way for comprehensive dental enhancements.
